# Subglottal pressure oscillations in anechoic and resonant conditions and their influence on excised larynx phonations

**DOI:** 10.1038/s41598-020-79265-3

**Published:** 2021-01-08

**Authors:** Sarah Lehoux, Vít Hampala, Jan G. Švec

**Affiliations:** Voice Research Lab, Department of Biophysics, Faculty of Science, Palacký University, 17. Listopadu 12, 771 46 Olomouc, Czechia

**Keywords:** Biological physics, Fluid dynamics

## Abstract

Excised larynges serve as natural models for studying behavior of the voice source. Acoustic resonances inside the air-supplying tubes below the larynx (i.e., subglottal space), however, interact with the vibratory behavior of the larynges and obscure their inherent vibration properties. Here, we explore a newly designed anechoic subglottal space which allows removing its acoustic resonances. We performed excised larynx experiments using both anechoic and resonant subglottal spaces in order to analyze and compare, for the very first time, the corresponding subglottal pressures, electroglottographic and radiated acoustic waveforms. In contrast to the resonant conditions, the anechoic subglottal pressure waveforms showed negligible oscillations during the vocal fold contact phase, as expected. When inverted, these waveforms closely matched the inverse filtered radiated sound waveforms. Subglottal resonances modified also the radiated sound pressures (Level 1 interactions). Furthermore, they changed the fundamental frequency (*f*_*o*_) of the vocal fold oscillations and offset phonation threshold pressures (Level 2 interactions), even for subglottal resonance frequencies 4–10 times higher than *f*_*o*_. The obtained data offer the basis for better understanding the inherent vibratory properties of the vocal folds, for studying the impact of structure-acoustic interactions on voice, and for validation of computational models of voice production.

## Introduction

The well-known source-filter theory proposed by Gunnar Fant^1^ described the voice production mechanism as a sound source (the exhalatory air flow modulated by vocal fold vibrations), filtered by acoustic resonances in the supraglottal cavities above the vocal folds (i.e. vocal tract resonances), and supposed no interaction between the source and the filter. Although this theory works well for speech analysis and synthesis, it is not sufficient to explain several voice production phenomena, such as vocal fold self-oscillations and voice instabilities. Deeper insights into the mechanism of the self-sustained vocal fold oscillation are provided through the myoelastic-aerodynamic (MEAD) theory of voice production formulated by van den Berg^2^ and further elaborated by Titze^3–5^. This theory predicts interactions between the vocal fold vibrations and the surrounding pressures (i.e., subglottal and supraglottal pressures), leading to interdependency and nonlinear phenomena^6–8^. Titze categorized the source-filter interactions in two levels: Level 1 interactions exhibit changes in the source flow waveform; Level 2 interactions exhibit changes in the vocal fold oscillations^9^. Observations on the interaction phenomena preceded the MEAD theory: as early as in 1932, D. Weiss reported on singing voice instabilities induced by adding a resonance tube to the vocal tract^10^. Later, e.g. Titze et al.^11^, Wade et al.^12^ and Zañartu et al.^13^ reported on occurrences of sudden pitch frequency jumps and other instabilities when the fundamental frequency of oscillation *f*_*o*_ was in the vicinity of the first vocal tract resonance frequency. These phenomena were also observed through numerical simulations by several authors^9,14–17^. Interactions with subglottal resonances might have a similar influence on the voice source waveform and the vocal fold vibrations as the vocal tract, as observed by Austin et al.^18^ (using an excised larynx), Zhang et al.^19,20^ (using vocal fold physical models and an excised larynx), or Lucero et al.^21^ (using vocal fold physical and mathematical models).

Excised larynges allow obtaining deeper insight into the natural behavior of the voice source as their properties closely approximate those of the living larynges. Acoustic resonances inside the air-supplying tubes below the larynx (i.e., subglottal space), however, may influence the vibratory behavior and obscure the inherent vibratory properties of the larynx. To the best of our knowledge, no data exist revealing on how much the laryngeal vibratory behavior differs between anechoic and resonant subglottal conditions. In this paper, we therefore explore excised larynges with a newly developed anechoic (resonance-free) subglottal tract^22^. This setup eliminates the acoustic interactions with both the vocal and subglottal tracts and allows studying the vibration properties of the vocal folds in their inherent state. We measured the acoustic response of the newly developed anechoic tract and compared it to the acoustic response of an adjustable “resonant” subglottal tract, previously developed by Hampala et al.^23^. Finally, we used these subglottal tracts in excised larynx experiments where we measured the subglottal pressure waveforms and the radiated sound. The vocal fold vibrations were simultaneously monitored by the electroglottographic (EGG) signal, which is an approximate measure of the changes in the vocal fold contact area^24,25^. In these experiments, we compared the subglottal pressure waveforms and investigated the influence of the subglottal acoustics on the vocal fold vibrations and on the radiated sound.

## Results

### The acoustic responses of the subglottal tracts

**Figure 1:**
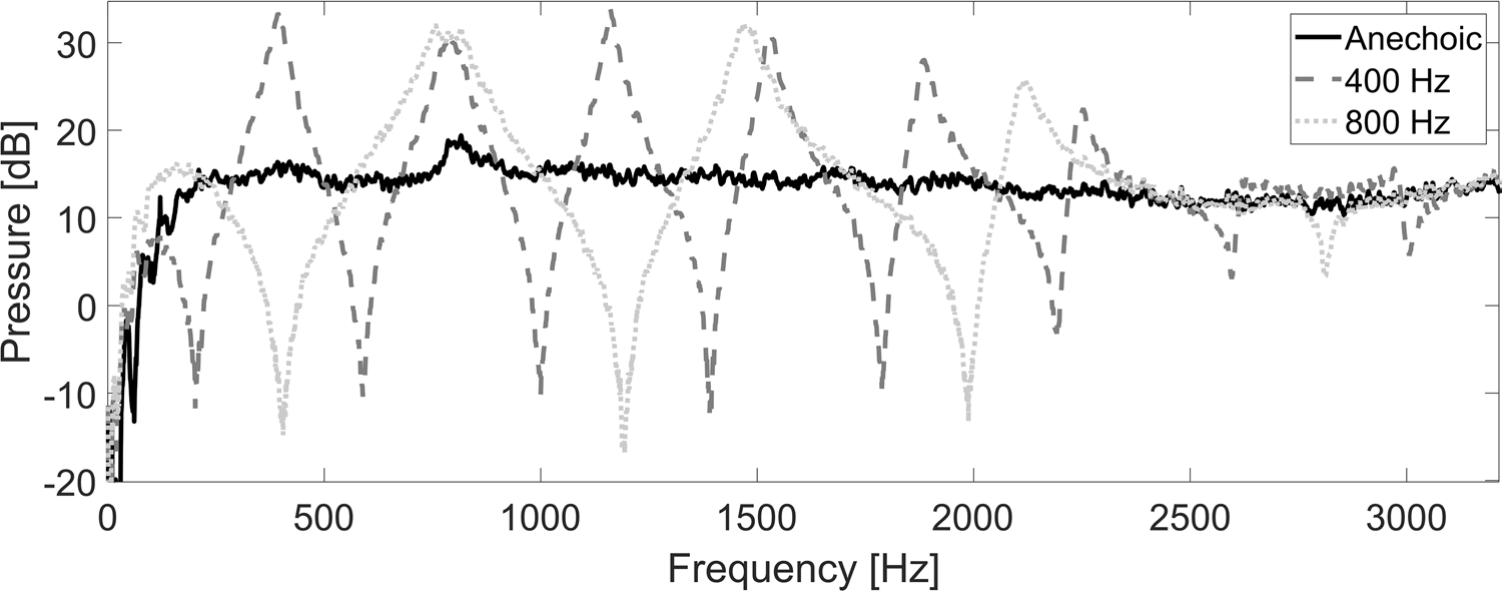
Frequency responses of the anechoic subglottal tract and of the resonant subglottal tract with the piston set to two different positions corresponding to *f*_*R1*_ = 400 and 800 Hz. Whereas the resonant tracts show multiple resonances and antiresonances (as expected for a tube with closed ends), the anechoic tract is practically free of these.

To verify the functionality of the anechoic and resonant subglottal tracts we first measured their frequency responses. Figure 1 shows the responses for the anechoic subglottal tract and for the resonant subglottal tract set to two different resonance frequencies: *f*_*R1*_ = 400 and 800 Hz (only two resonance settings are presented here for simplicity). The resonant subglottal tract exhibits clear resonances and anti-resonances, as expected from the straight circular waveguide approximation: the lowest resonances and anti-resonances follow the patterns *f*_*Rn*_ = n*f*_*R1*_ and *f*_*ARn*_ = (2n—1)*f*_*AR1*_, where n is a positive integer. Importantly, the anechoic tract appears free of the acoustic resonances and has a response similar to one of an infinite, purely resistive waveguide. A small peak around 800 Hz was present in all the frequency responses, we therefore concluded that it was related to the damped resonance inside the air supply tubes.

### Excised larynx experiments

#### Pressure and EGG waveforms in the anechoic and resonant conditions: steady phonations

**Figure 2:**
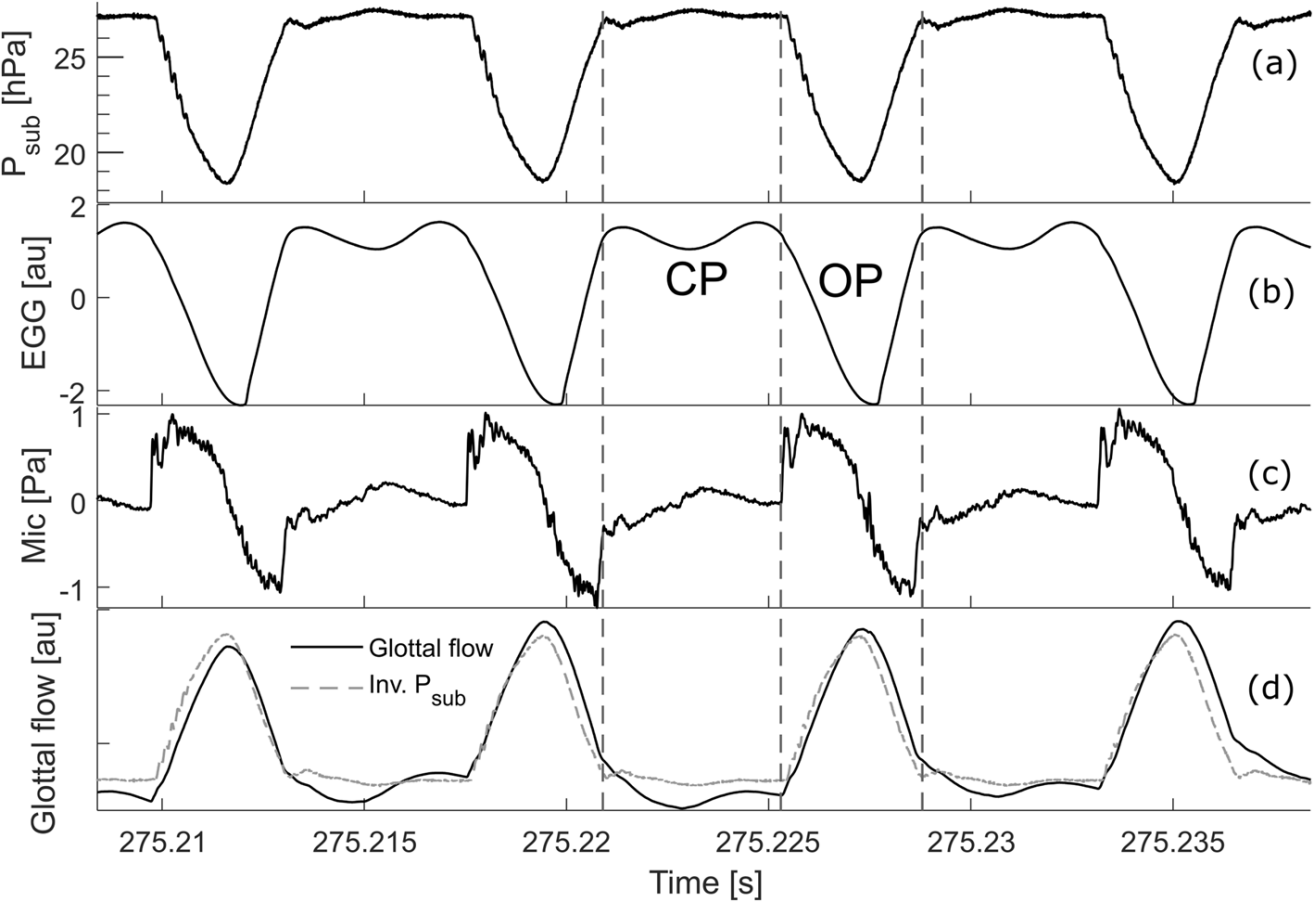
Signal waveforms for the excised larynx experiment in steady phonation, obtained using the anechoic subglottal tract. (**a**) Subglottal pressure; (**b**) EGG signal in arbitrary units [au]; (**c**) microphone signal (radiated sound) at 10 cm distance from the glottis; (**d**) glottal flow signal obtained by inverse filtering (black solid line) and inverted subglottal pressure (dashed gray line). The inverted subglottal pressure waveform in (**d**) was scaled to match the amplitude of the flow to observe the similarity of these waveforms. The closed phase (CP) and open phase (OP) were identified manually using both EGG and subglottal pressure signals.

To find out the effect of the anechoic and resonant subglottal tracts in excised larynx phonations we studied the subglottal pressure signal waveforms, detected with a pressure sensor just below the vocal folds. Those signals were obtained during steady phonations where the mean subglottal pressure had attained a saturation value, and the mean flow was set constant to about 400 mL s^−1^. Figure 2 shows the subglottal pressure (a), EGG (b) and microphone (c) signal waveforms recorded while using the anechoic subglottal tract. The dashed vertical lines show the approximate instants of closure and opening of the vocal folds which were identified based on the subglottal pressure and EGG signal waveforms. As the anechoic subglottal tract is effectively equivalent to an infinite, purely resistive waveguide, it is expected that the pressure inside this waveguide is proportional to the flow^26^. Indeed, the subglottal pressure waveform resembled an inverted theoretical glottal flow voice source signal^27–32^: it was approximately constant during the closed phase, decreasing during the opening phase and increasing during the closing phase (Fig. 2a). To observe the similarity of the subglottal pressure waveform to the glottal flow signal, we performed inverse filtering analysis of the microphone signals. For this, we used the numerical integration feature offered by the Sopran software developed by Svante Granqvist^33^, simulating the radiation impedance without any vocal tract. As expected, the resulting waveform was almost identical to the inverted subglottal pressure waveform obtained using the anechoic subglottal tract (Fig. 2d). The inverse filtered waveform was slightly more perturbed than the anechoic subglottal waveform, however.

**Figure 3:**
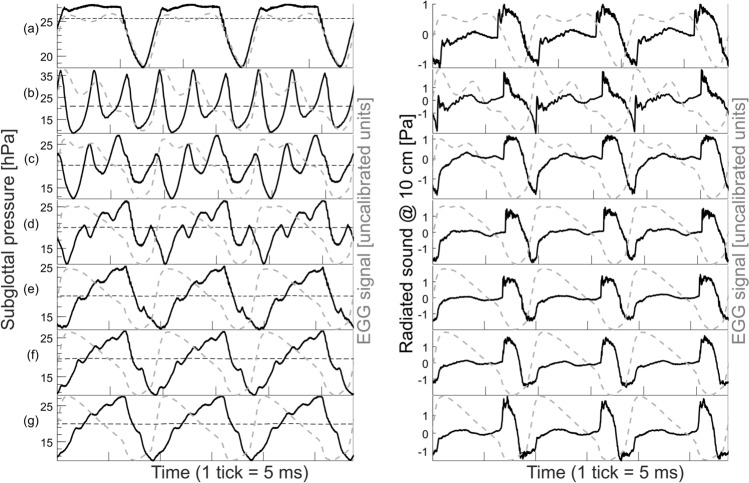
Subglottal pressure (left, solid black lines) and radiated sound (right, solid black lines) waveforms registered with an anechoic subglottal tract and with resonant subglottal tracts set to different resonance conditions: (**a**) anechoic subglottal tract, (**b**) *f*_*R1*_ = 330 Hz, (**c**) *f*_*R1*_ = 400 Hz, (**d**) *f*_*R1*_ = 500 Hz, (**e**) *f*_*R1*_ = 600 Hz, (**f**) *f*_*R1*_ = 700 Hz and (**g**) *f*_*R1*_ = 800 Hz. The horizontal dashed black lines on the left panel indicate the mean subglottal pressure in each case. The waveforms are individually scaled in time (3 cycles are shown). The instants of closure and opening were approximately synchronized using the corresponding EGG signal waveforms (dashed gray lines). Notice the drastic difference in the subglottal pressure waveforms between the anechoic (**a**) and resonant (**b**–**g**) cases. In (**b**–**g**), notice the increased frequency of the secondary subglottal oscillations related to the increasing resonance frequency of the subglottal tract. The radiated audio signals also show changes with the different subglottal tract conditions but these are smaller compared to those of the subglottal pressure and occur mainly during the glottal open phase.

In contrast to the anechoic tract, the subglottal pressure waveform appeared more complex when using the resonant subglottal tract. This is shown in Fig. 3 which compares the subglottal pressure waveforms (left, black solid lines) and the radiated sound waveforms (right, black solid lines) for the anechoic (Fig. 3a) and resonant subglottal tract set to six different resonance frequencies: *f*_*R1*_ = 330, 400, 500, 600, 700 and 800 Hz (Fig. 3b–g). These signals were scaled in time to show exactly three cycles for each signal. The glottal opening and closing instants were approximately synchronized using the corresponding EGG waveforms (grey dashed lines). In contrast to the anechoic subglottal pressure waveforms which showed very little fluctuations during the glottis closed phase, the presence of subglottal acoustic resonances introduced fluctuations of the subglottal pressure during the closed phase similarly as observed previously in vivo^34–37^ (see Fig. 3b–g, left). The frequency of the subglottal pressure fluctuations increased when *f*_*R1*_ increased, further indicating that they are caused by acoustic resonances in the subglottal tract. For the constant flow of 400 mL∙s^−1^ the mean subglottal pressures (horizontal dashed black lines in Fig. 3, left) were about 25 hPa (i.e. c. 25 cm H_2_O) in the anechoic conditions, whereas they were between 19 and 21 hPa in the resonant conditions. The radiated sound waveforms also showed changes with the different subglottal tract conditions (see Fig. 3, right), revealing that the subglottal tract influences also the radiated sound and thus indicating the presence of Level 1 interactions. These waveform changes were smaller compared to those of the subglottal pressure waveforms, however. For lower subglottal resonance frequencies, the EGG waveforms exhibited secondary peaks which approximately coincided with the peaks of the subglottal pressure waveforms (e.g. Fig. 3b,c). This suggests that the subglottal resonances influenced also the vocal fold vibrations, indicating Level 2 interactions. For higher subglottal resonance frequencies (Fig. 3d–g), the secondary EGG peaks did not occur and the EGG waveforms appeared nearly identical. Interestingly, the fundamental frequency of the vocal fold oscillations was found lowered in the resonant conditions (*f*_*o*_ around 106 Hz) compared to the anechoic ones (*f*_*o*_ around 126 Hz), even though the laryngeal settings were kept constant. The change of *f*_*o*_ between anechoic and resonant conditions suggests the presence of Level 2 interactions. Surprisingly, however, the *f*_*o*_ stayed around 106 Hz and did not change when the subglottal tract setting was changed among the six different resonance frequencies.

**Figure 4:**
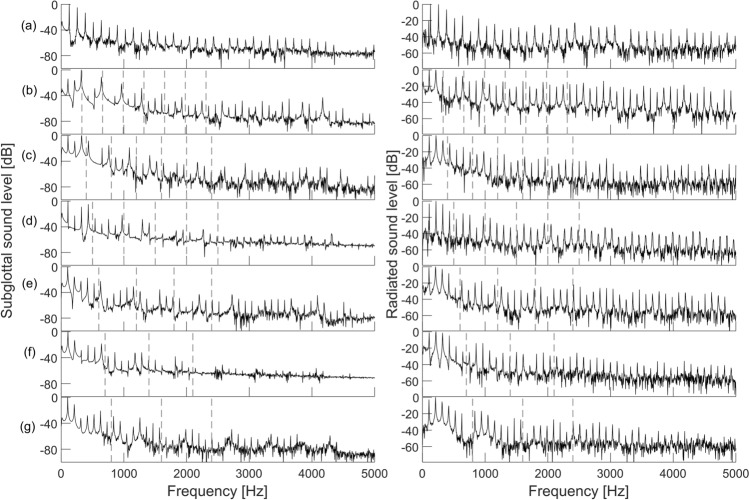
Spectra of the subglottal (left) and radiated (right) sounds, corresponding to the waveforms shown in Fig. 3. The subglottal settings were: (**a**) anechoic subglottal tract, (**b**) *f*_*R1*_ = 330 Hz, (**c**) *f*_*R1*_ = 400 Hz, (**d**) *f*_*R1*_ = 500 Hz, (**e**) *f*_*R1*_ = 600 Hz, (**f**) *f*_*R1*_ = 700 Hz and (**g**) *f*_*R1*_ = 800 Hz. The vertical dashed lines indicate the subglottal resonance frequencies up to 2.5 kHz, measured with no larynx attached. The spectra were produced at the frequency resolution of 4 Hz and were normalized by calculating the maximum value in each spectrum and setting it to 0 dB.

The spectra of the waveforms from Fig. 3 are presented in Fig. 4. Again, the subglottal spectra are on the left and the radiated spectra on the right. Clear harmonic components appeared in all the spectra at multiples of the fundamental frequency, revealing that the oscillations occurred at a steady pitch. The resonant subglottal spectra (Fig. 4b–g, left) exhibited a repetitive formant structure, reflecting the resonances and antiresonances observed in the frequency response of the resonant subglottal tracts (recall Fig. 1). The frequency of the first formant and the distance between the formants increased with the increasing resonance frequencies of the subglottal tract, as expected. The formant frequencies were, however, slightly lower than the resonance frequencies previously measured on the subglottal tracts without the larynx (indicated by dashed vertical lines in Fig. 4b–g). This difference can be explained by changes in the boundary conditions caused by the larynx. As expected, the envelope of the anechoic subglottal spectrum (Fig. 4a, left) was more uniform than in the resonant conditions and did not display a clear repetitive formant structure. The amplitude of the harmonic components decreased here with increasing frequency, although some fluctuations of the spectral envelope were also present.

The radiated sound spectra are shown in Fig. 4 on the right. In contrast to the resonant subglottal spectra, they did not display such a prominent formant structure in the resonant conditions. Nevertheless, slight modulation of the envelope of the radiated spectra occurred here too and the distances between the spectral envelope maxima appeared to increase with the increasing resonance frequency of the subglottal tract (Fig. 4b–g, right) similarly as in the subglottal spectra. This suggests that the subglottal resonances partially transferred to the radiated spectra. However, the subglottal resonance peaks were very broad and much less distinctive there. The radiated spectrum for the anechoic case (Fig. 4a, right) also showed some fluctuations in its envelope. It differed slightly from the radiated resonant spectra but these differences were, again, less distinct than the differences between the anechoic and resonant spectra in the subglottal space. As expected, the slope of the harmonic decay (the decrease in the peak amplitude for every doubling of the frequency) appeared smaller in the radiated sound spectra than in the subglottal sound spectra. This can be explained by the high-frequency amplification caused by the sound radiation into free air (see Eqs. (3) and (4) in the appendix).

#### Phonation changes due to interactions with the subglottal acoustics: flow sweeps

**Figure 5:**
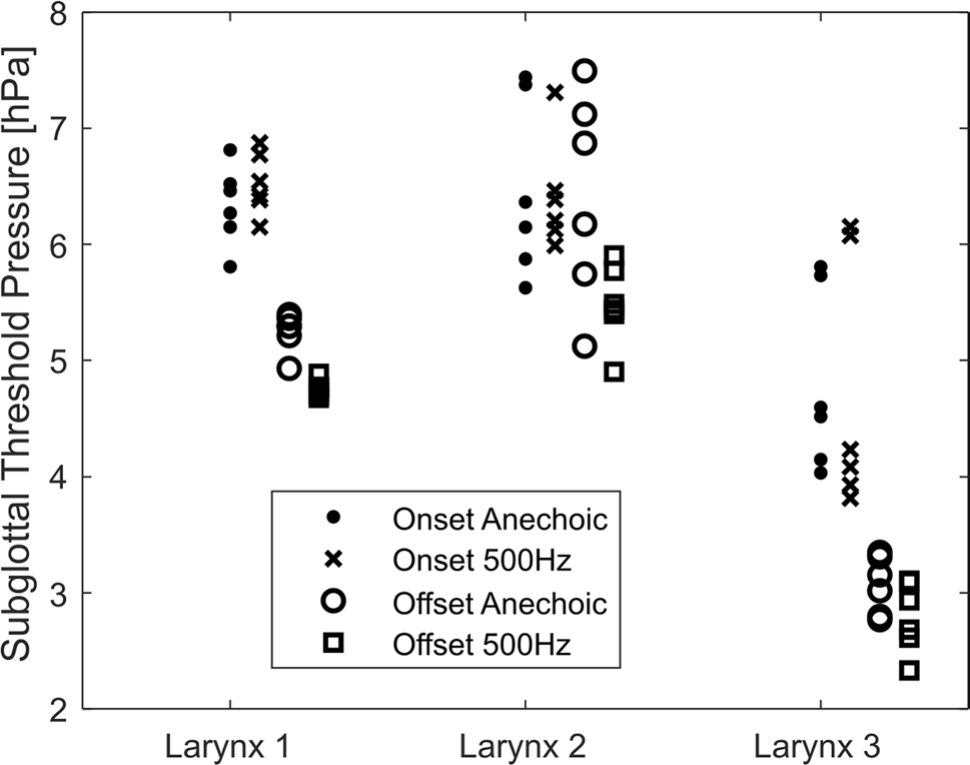
Oscillation threshold pressure for each larynx with an anechoic subglottal tract and resonant tract set to 500 Hz subglottal resonance. Each point corresponds to one flow sweep. Notice the onset thresholds are generally higher than the offset thresholds. The subglottal acoustics had little influence on the onset pressures (dots versus crosses), but the offset pressures were significantly lower for the resonant subglottal tract (circles versus squares). For the numerical values and the statistical results, see the Supplementary material S1 to this article.

In order to find out whether there was an influence of the subglottal resonances on the vibrational properties of the vocal folds, we investigated the phonation threshold onset and offset pressures, the frequency of the vocal fold oscillations *f*_*o*_, and the SPL of the subglottal and the radiated sounds. We analyzed the data from repeated flow sweeps, where the flow was slowly increased to about 550 mL∙s^−1^ and slowly decreased back to zero. The experiments were done with an anechoic tract and with a resonant tract set to 500 Hz subglottal resonance frequency. In both the anechoic and resonant conditions, the offset pressures were generally smaller than the onset pressures (see Fig. 5). This agrees with the theoretical studies of vocal fold oscillation onset and offset^38,39^ as well as with previous experimental observations on vocal fold mucosa^40^ and excised larynges^41–43^.

To find whether the subglottal resonance conditions had significant effect on the onset and offset phonation threshold values, we used multiple linear regression models (see the Supplementary material S1 for details on the statistics). The onset pressure values were not significantly different (p = 0.97) between the resonant and anechoic conditions. The offset pressure values were, however, approximately 11% lower (95% confidence interval 6–16%) in the resonant conditions than in the anechoic conditions, and this effect was statistically significant (p = 0.0003). These results suggest that the subglottal acoustics has little influence on the oscillation onsets, but has significant influence on the oscillation offsets.

**Figure 6:**
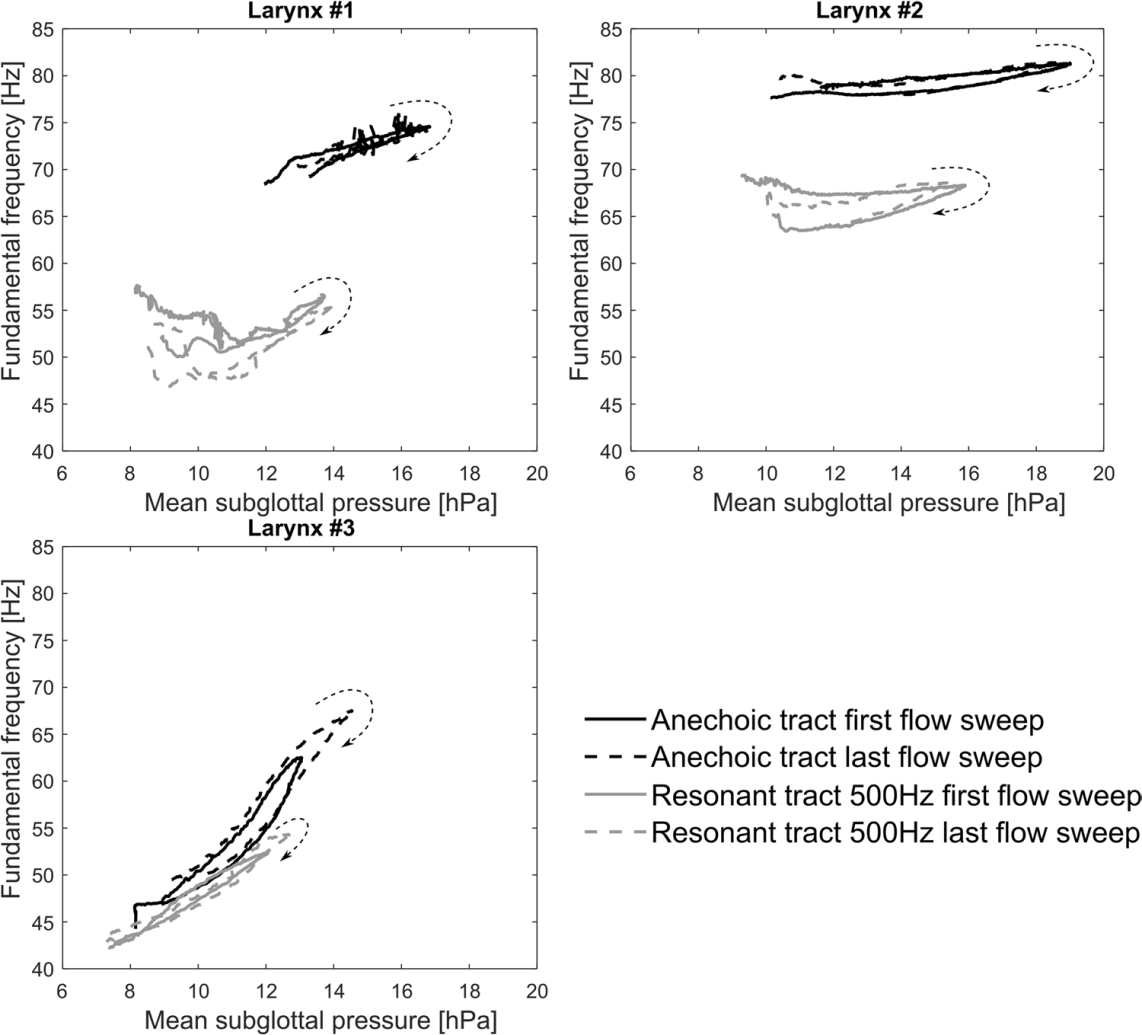
Analysis of *f*_*o*_ of the vocal fold oscillations with regards to the mean subglottal pressure. The results from the first (solid lines) and last (dashed lines) flow sweeps are showed, for each larynx. The black lines mark the data measured with the anechoic tract, whereas the gray lines mark the data measured with the resonant subglottal tract set to *f*_*R1*_ = 500 Hz. The dashed arrows indicate, for each flow sweep, the evolution of the measured values through time. In all the three larynges, the *f*_*o*_ values are lower for the resonant tract than for the anechoic one. The *f*_*o*_ differences vary among the three larynges, however.

Figure 6 shows the *f*_*o*_ values obtained from the phonations of the three larynges during the flow-sweep experiments. The *f*_*o*_ values were different for the different larynges. However the same phenomenon was visible in all the three larynges: the *f*_*o*_ values were consistently higher in the anechoic than in the resonant subglottal tract for the same subglottal pressures. This corroborates similar observation from the steady flow experiment where the *f*_*o*_ was also higher in the anechoic tract compared to all the resonant tracts. For clarity, only the values from the first and last of the flow sweeps are shown in Fig. 6, but these show the repeatability along the sweeps. Near the phonation onsets and offsets, the larynges exhibited irregular vocal fold vibrations, therefore we did not include these parts of the signal in the *f*_*o*_ analysis. Only the parts with a stable *f*_*o*_ were kept for analysis.

**Figure 7:**
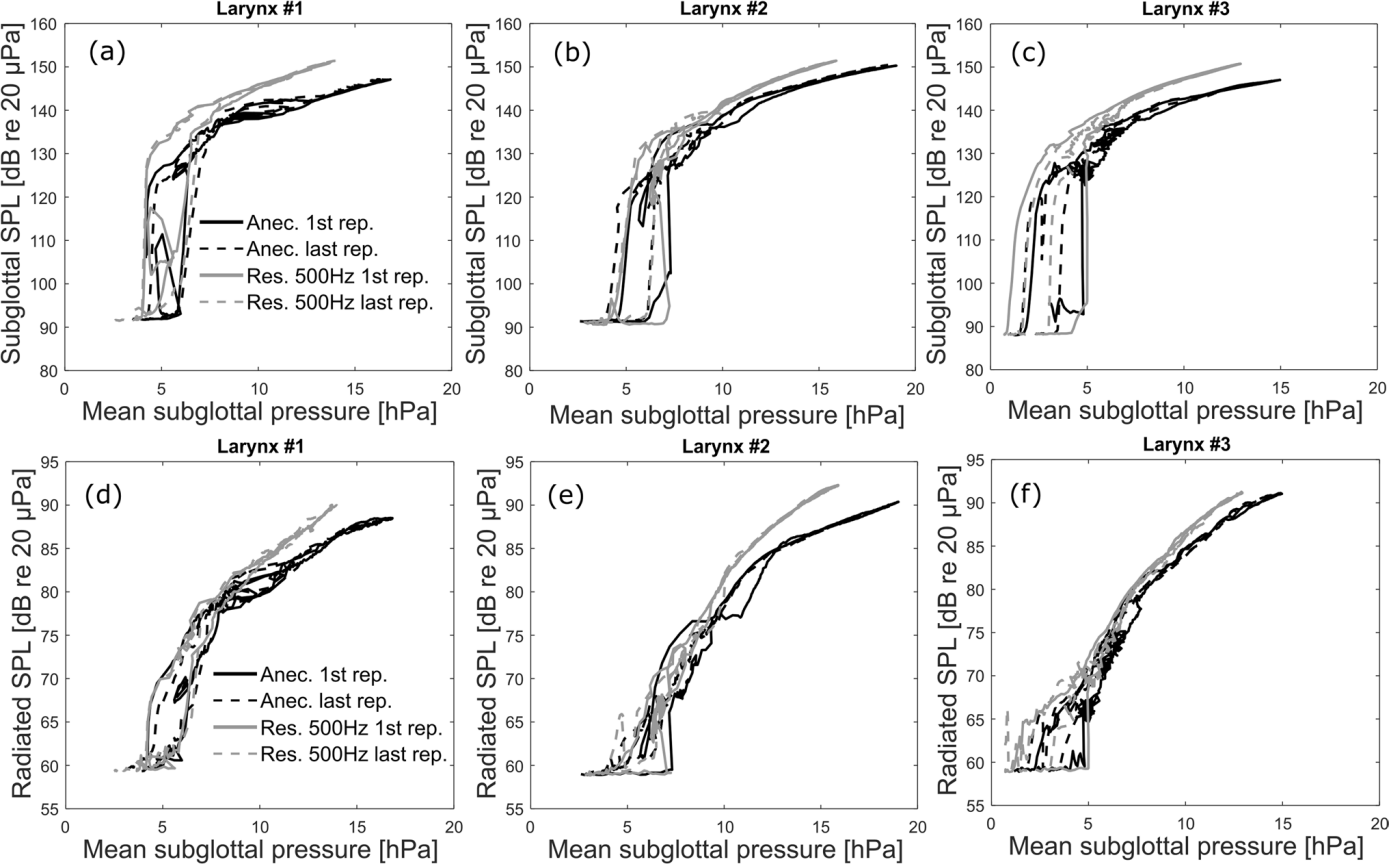
Analysis of the SPL (non-weighted) of the subglottal (**a**–**c**) and radiated (**d**–**f**) sounds at 10 cm distance with regards to the mean subglottal pressure. The black lines mark the data measured with the anechoic tract, whereas the gray lines mark the data measured with the resonant subglottal tract set to *f*_*R1*_ = 500 Hz. (**a**,**d**) First larynx, (**b**,**e**) second larynx, (**c**,**f**): third larynx. Notice that for a given subglottal pressure the SPLs are higher in the resonant than in the anechoic tract. For clarity, only the results from the first and last flow sweeps are shown, for each larynx. The solid lines correspond to the first flow sweep and the dashed lines to the last flow sweep for each larynx, illustrating the repeatability of the values.

For given mean subglottal pressures, the SPL of the subglottal sound was found to be higher when using the resonant tract, as shown in Fig. 7a–c. At the mean subglottal pressures above 8 hPa, the difference in SPL was about 6–8 dB for the first larynx, 2–3 dB for the second larynx and 4–5 dB for the third larynx. In both the anechoic and resonant cases the subglottal SPLs showed extremely high values reaching up to 150 dB re 20 µPa.

Compared to the subglottal SPLs, the SPLs of the radiated sound at 10 cm distance were about 60 dB (!) lower reaching the maximum values of about 90 dB re 20 µPa. Similarly to the SPL of the subglottal sound, the radiated sound showed higher values when using the resonant tract, for the same subglottal pressures, as demonstrated in Fig. 7d–f. This increase was especially visible for mean subglottal pressure values above approximately 800 Pa. The increase in SPL, for identical mean subglottal pressures above 8 hPa, was about 2.5–3.5 dB for the first larynx, 2–4.5 dB for the second larynx and 1.5–2.5 dB for the third larynx.

## Discussion

While subglottal resonances have been observed to influence voice and vocal fold vibrations^19^, hardly any experimental data have been available documenting the voice and laryngeal behavior when the subglottal resonances are not present. Yet, removing the interactions with acoustic resonances is important for understanding the inherent vibratory properties of the voice source and of the vocal folds, and for validating computational models of voice production. To the best of our knowledge, Zhang et al.^44^ did the first and so far the only study, which attempted to design and use an anechoic subglottal tract for voice generation. They used it for studying sound produced through an orifice simulating glottis with time-varying area. Anechoic terminations consisting of two connected perforated rubber hoses sealed with fiberglass were inserted into the air-supplying tube. Measurements of the frequency response revealed reduction of the subglottal resonances to some extent, although not fully. No excised larynx experiments using this tract were reported in the study.

In our experiments, we used a newly developed anechoic subglottal tract with a different design^22^. The measured frequency response (recall Fig. 1) revealed that the newly developed anechoic subglottal tract was able to cancel most of the acoustic resonances in the subglottal space. In this respect, the new design appears to be more effective than the anechoic subglottal tract pioneered by Zhang et al.^44^. Furthermore, the adjustable resonant subglottal tract allowed creating and changing the subglottal resonances to study their influence on the voice source and vocal fold behavior. The resonant subglottal tract allowed us to modify the lowest subglottal resonance frequencies from c. 330 to 800 Hz. This covers the range of subglottal resonance frequencies in humans, which are expected to be between 500 and 700 Hz^45–47^. The vocal fold fundamental frequency of 100–120 Hz measured in the red deer larynx during the steady phonation experiment corresponds to low-pitched phonations of male human subjects. The fundamental frequencies in the flow sweep experiment were lower, however, around 40–80 Hz, because larger red deer larynges were used.

To our knowledge, this study is the first to directly demonstrate the effect of absence and presence of subglottal resonances on phonation properties of excised larynges. Excised larynges are considered to be the most representative models of living vocal apparatus, therefore the observed effects can be expected to be similar to those observed in vivo. Our results clearly indicate that subglottal resonances influence both the radiated acoustic signals, as well as the vocal fold oscillations. Overall, for subglottal resonance frequencies that were much higher than the fundamental frequencies of vocal fold oscillations, their presence was found to slightly change the shape of the radiated acoustic waveforms (Fig. 3) and increase the radiated sound pressure levels by up to c. 4.5 dB (Fig. 7). The spectrum of the radiated sound showed some differences among the anechoic and resonant subglottal conditions. These differences were, however, considerably less apparent than those in the subglottal sound spectra which showed prominent formants in the resonant conditions (recall Fig. 4). The changes of the radiated pressure waveform, its SPL and spectrum belong to Level 1 interactions, but they can be influenced also by the Level 2 interactions as discussed below.

The presence of subglottal resonances was found also to change the fundamental frequency of the vocal fold oscillations (Fig. 6) and the threshold pressure for phonation offset (Fig. 5). These differences indicate the occurrence of Level 2 structure-acoustic interaction^9^, as a change of subglottal acoustics induces a change in the vocal fold vibrations. Interestingly, no clear influence of subglottal resonances was found for the threshold pressure in phonation onset (Fig. 5). This suggests that the mechanisms for voice onset and offset should be seen differently from the perspective of subglottal interactions: subglottal resonances can be expected to be little excited in both the anechoic and resonant tracts before the phonation starts, thus having little influence on the phonation onset. At voice offset, however, the subglottal resonances are excited in a resonant tract, influencing the offset differently than the anechoic tract where no resonances occur.

The subglottal pressure oscillation amplitudes shown in Fig. 3 were around 800 Pa in the anechoic case and about twice as much in the resonant cases. These pressure fluctuations correspond to extremely high sound pressure levels around 140–150 dB re 20 µPa (non-weighted) which are indicated in Fig. 7. Such strong subglottal pressure oscillations appear to influence also the vocal fold tissue oscillations causing changes in the electroglottographic waveform (shown in Fig. 3), again indicating Level 2 interactions. Subglottal pressure oscillations of similar magnitude were observed also in the in vivo human data^34,36,37,48,49^. The increase of the subglottal SPL in the resonant case (shown in Fig. 7a–c) is likely caused by the presence of subglottal acoustic resonances, which are boosting some harmonics of the source signal (see Fig. 4b–d, left). It is also possible that the vocal folds vibrated with more amplitude when using the resonant subglottal tract, which would also be a Level-2-interaction effect, but verifying this assumption would require accompanying laryngeal video recordings which were not available for these pilot experiments.

**Figure 8:**
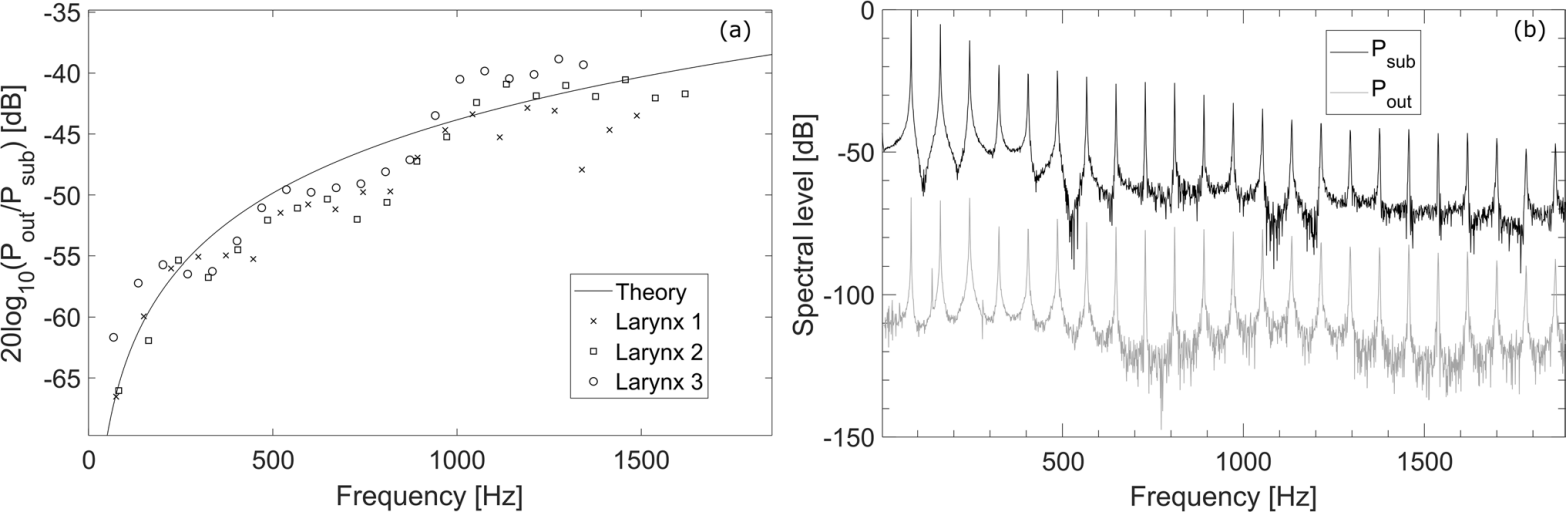
(**a**) Ratios of the first 20 spectral peak amplitudes between the radiated sound pressure and the subglottal pressure, expressed in dB, for the three larynges used during the flow sweeps experiment with anechoic subglottal tract. The solid line shows the expected ratio values according to the theory described in the appendix (value $A_{L}$ from Eq. 5). (**b**) Example spectra of the subglottal (black line) and radiated (gray) sound pressures in the anechoic conditions. These spectra were taken from the second larynx, obtained from a one-second window at the maximum mean subglottal pressure during the first flow sweep, and normalized to the value of the first peak in the subglottal sound pressure spectrum.

The radiated sound levels at 10 cm distance were about 60 dB lower than the subglottal sound levels. The highest radiated sound levels achieved here were around 90 dB (non-weighted) whereas the highest subglottal SPL were around 150 dB. The approximate 60 dB difference is a consequence of the conversion of the subglottal acoustic pressures to the acoustic volume flow which serves as the acoustic source radiating the sound to the surrounding space. The theoretical relationship between the subglottal and radiated sound pressures is derived in the Appendix. The radiation impedance for the glottal sound source is frequency dependent, therefore the low-frequency spectral components radiate less efficiently than the high-frequency components. Figure 8a compares the theoretical relationship between the subglottal and radiated pressures to the corresponding experimentally observed values in our excised larynx flow-sweep experiments. The values were obtained by comparing the spectral harmonic components of the subglottal and radiated sound (Fig. 8b). Even though the model relies on simplifying assumptions, the experimental and theoretical values follow the same trend and match reasonably well. The final SPL difference between the subglottal and radiated sound depends mainly on the dominant components of the sound spectrum which are around the lowest harmonic frequencies^50^. In our case these were around 50–200 Hz (Fig. 8b) where the decrease is, indeed, around 60 dB (Fig. 8a).

In the steady flow experiment shown in Fig. 3, we noticed that the mean subglottal pressures were higher for the anechoic than for the resonant conditions, in contrast to the oscillatory pressure amplitudes which showed an opposite tendency. Similarly, in the flow sweep experiments higher mean subglottal pressures were achieved in the anechoic conditions for the highest flows, as seen in Fig. 7. This suggests that glottal resistance is higher in anechoic than in the resonant conditions. Since the vocal fold settings were kept constant throughout the experiments, this is likely related to altered vocal fold oscillations (again indicating Level 2 interaction), possibly to their larger vibratory amplitudes in resonant conditions, but finding more about this effect would again require high-speed videolaryngoscopic data which were not available for these experiments. We plan to address this issue in future studies.

In an experiment with synthetic vocal folds and modifiable subglottal tract resonances, Zhang et al.^19^ reported on a strong tendency of the vocal fold *f*_*o*_ to be driven towards the first, second, third or fourth subglottal resonance frequency. In our flow sweep experiments, we avoided such effects by keeping the subglottal resonances well above, i.e. 6–10 times higher than the vocal fold *f*_*o*_, analogously as it is in the low-pitched phonations in humans. Interestingly, even under these conditions the presence of the subglottal resonances caused the vocal fold *f*_*o*_ to be decreased compared to anechoic conditions in all the cases investigated (Fig. 6). The amount of *f*_*o*_ change was different for the different larynges, however (Fig. 6). Once the resonant subglottal tract was attached, changing its resonance frequency between 330 and 800 Hz did not cause any significant change of *f*_*o*_ in the steady-flow-excised-larynx experiment (Fig. 3). The *f*_*o*_ remained to be around 106 Hz here. This suggests that no strong resonance tuning effects occurred between the subglottal resonances and the vocal fold oscillations. Nevertheless, the *f*_*o*_ differences between the anechoic and resonant subglottal conditions indicate that the mere presence of the resonances in the subglottal system can influence the vocal fold vibrations (Level 2 interaction). In future, it is desirable to confirm these effects also on human larynges.

The anechoic subglottal tract offers an interesting possibility to get direct information on the voice source waveform. As shown in Fig. 2, the vertically inverted subglottal pressure waveform matches well the inverse-filtered acoustic waveform. It shows the signal increasing and decreasing when the vocal folds open and close and being relatively constant during the glottis closure, as expected by the source-filter theory^51,52^. Compared to the subglottal-pressure-based waveform, the inverse-filtered waveform from the radiated sound shows more perturbations, suggesting the radiated sound is more polluted by surrounding noise and sound reflections from the structures around the larynx. The cleaner anechoic subglottal pressure waveform therefore appears to be advantageous for monitoring the voice source and can be explored in future studies.

## Conclusion

The newly developed anechoic subglottal tract successfully removed its acoustic resonances to suppress their influence on vocal fold vibrations. When used in excised larynx experiments, the subglottal acoustic pressure waveform was similar to the inverted glottal flow source signal: almost constant during the closed phase, decreasing during the opening phase and increasing during the closing phase. In comparison, when using a resonant subglottal tract, the subglottal pressure exhibited fluctuations during the closed phase, which were related to the subglottal acoustic resonances, as expected. The subglottal resonances were found to influence both the radiated acoustic waveforms and radiated sound pressure levels, as well as the vocal fold oscillations. These provide direct evidence of the occurrence of both the Level 1 and Level 2 interactions of the voice source with subglottal pressure oscillations. The developed anechoic subglottal tract can be used to study the inherent properties of the voice source and vocal fold oscillations, free of acoustic interactions with adjacent cavities. The obtained data offer the basis for better understanding the inherent vibratory properties of the voice source, for studying the impact of structure-acoustic interactions on voice source, and for validation of simulation results obtained from computational models of voice production.

## Methods

### Design of the anechoic and resonant subglottal tracts and excised larynx experiments setup

**Figure 9:**
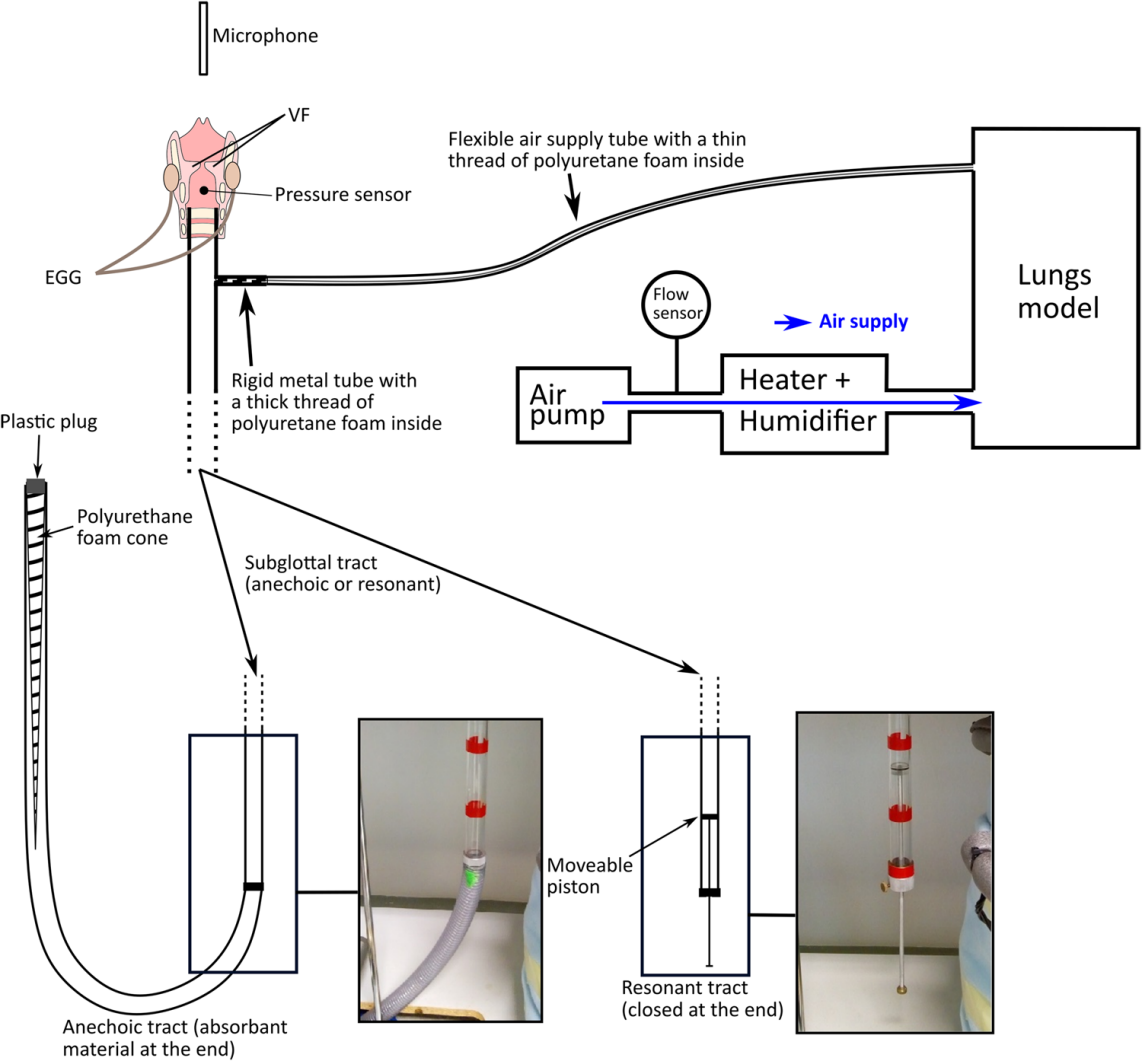
Excised larynx experimental setup.

The design of the subglottal tract was shortly described in a preliminary conference paper^22^. Here we provide its complete description. When designing the subglottal tract, we divided the subglottal spaces into a primary subglottal space and a secondary air-supply system. The primary subglottal space consisted of a 55 cm long straight cylindrical Plexiglas tube with the diameter of 24 mm. This tube formed an air space which could be changed from resonant to anechoic. Figure 9 shows simplified drawings and photographs of the subglottal tract in resonant and anechoic mode. In the resonant case, the tube was terminated by a piston the position of which could be changed to modify the resonance frequency of the subglottal space (Hampala et al.^23^). To change the resonant subglottal tract to the anechoic one, the piston was removed and the subglottal Plexiglas tube was extended by a plastic tube of the same diameter with the length of 330 cm terminated by a sound-absorbing pyramidal wedge. The wedge was approximately 200 cm long, and was made out of polyurethane foam (Molitan T-2337), with a density of about 23 kg∙m^−3^. We closed this end of the tube with a plastic plug in order to prevent air leaks and pressure drops (see Fig. 9). Our subglottal tract is then seen by the voice source as a virtually infinite waveguide. The design of the sound-absorbing polyurethane wedge was inspired by the work of Sondhi (1975) who used similar approach to create an anechoic vocal tract for the purpose of inverse filtering^27^.

The primary subglottal tract comprised a small side hole allowing the connection to the air-supply system using a small, rigid metal tube and then a long, flexible plastic tube. To prevent sound from entering or exiting the air supply system, the rigid metal tube was filled with polyurethane foam and a thinner thread of this foam (about 1 cm^2^ cross-sectional area) was inserted in the flexible plastic tube (see Fig. 9). The thread was used to absorb the noise generated by the air pump and to cancel acoustic resonances inside the air-supply tubes as much as possible, in order to prevent any acoustic interaction with the larynx. The tubes had a 15 mm diameter.

An air pump (RESUN LP 100) was used to generate a continuous airflow through the system. The airflow was heated and humidified, before going through an artificial lung model (an air tank with a volume of 50 L with an inserted acoustically absorbing polyurethane foam) and to the subglottal tract via narrow air supply tubes. We used a mechanical flow meter placed just after the pump to adjust and monitor the amount of flow going out of the pump. The flow was also measured with a flow sensor placed after the flow meter. During the experiments, an electroglottograph (EGG) device (Glottal Enterprises EG2-PC) registered the vocal fold contact area, using two small electrodes screwed to the sides of the thyroid cartilage. We registered the subglottal acoustic pressure with a 2.4 mm diameter pressure transducer (Kulite XCQ093) placed at the inner side of the subglottal wall through a hole in the dorsal ridge of the cricoid cartilage. This pressure transducer is sensitive to frequencies between 0 and 150 kHz, allowing to measure both the DC pressure and the AC acoustic signals. The pressure transducer was inserted into a warmed metal tube to prevent moisture condensation around the transducer. A condenser microphone (MicW M416) placed approximately 10 cm above the glottis registered the radiated sound. All signals were sampled at 200 kHz using a DEWE-43 USB data acquisition system and recorded in the associated software Dewesoft X2.

### Acoustic measurements

As the resonant subglottal tract has a shape of a simple straight circular waveguide, we expected it to exhibit harmonic resonance frequencies *f*_*Rn*_ = n*f*_*R1*_, where *f*_*R1*_ is the first resonance frequency and *f*_*Rn*_ the *n*^*th*^ resonance frequency. Anti-resonances were also expected to be present, following the pattern *f*_*ARn*_ = (2n—1)*f*_*AR1*_, where *f*_*AR1*_ is the first anti-resonance frequency and *f*_*ARn*_ the *n*^*th*^ anti-resonance frequency. We carried out pilot acoustic measurements to find the value of *f*_*R1*_ when the piston is at the lowest and highest positions, and to locate piston positions corresponding to ‘round’ resonance frequencies (400 Hz, 500 Hz, etc.). When doing this, we placed a small extension tube (about 3 cm long) at the upper open end of the subglottal system in order to approximate the space generally added by an excised larynx. To this extension tube, we attached a small electret microphone (AV-JEFE, TCM14) and a small loudspeaker (Ekulit LSF-23 M/N/G), using plasterine which enclosed the upper end to prevent air leaks. We used Audacity software (version 2.3.0)^53^ to generate a ten-second linear chirp from 50 to 5000 Hz, to play it through the loudspeaker and to record the response of the subglottal tract captured by the microphone. Finally, we used the spectrum-plotting feature from Audacity to quickly find the approximate value of *f*_*R1*_, by locating the first peak in the spectrum. We observed that *f*_*R1*_ was about 330 Hz when the piston was at the lowest position and about 800 Hz when the piston was at the highest position. We marked approximate positions of the piston corresponding to *f*_*R1*_ = 400, 500, 600 and 700 Hz with adhesive tape. These values were measured without a larynx specimen and might vary slightly when attaching one. The lengths of the subglottal tract corresponding to those values of *f*_*R1*_ were as follows: 46.5 cm (400 Hz), 37 cm (500 Hz), 31 cm (600 Hz) and 27 cm (700 Hz). As stated, the maximum length of the resonant subglottal tract was 55 cm (corresponding to *f*_*R1*_ = 330 Hz) and the minimum length was 25 cm (corresponding to *f*_*R1*_ = 800 Hz).

Consequently, we measured the acoustic responses of both subglottal tracts accurately using the same electret microphone and loudspeaker attached to the upper open end of the system. Again, we used plasterine to ensure the microphone and the loudspeaker were tightly fixed to the tract tube and to prevent air leaks. We used the following protocol to measure the acoustic response of both the anechoic and resonant subglottal tracts:

1. The loudspeaker played hundred impulses at the rate of one impulse per second. The impulse signal was manually generated by Audacity software.
2. The microphone registered the temporal response of all these impulses. The microphone signal was digitalized using a Focusrite Scarlett 2i2 2nd Gen USB audio interface and recorded by Audacity.
3. We segmented the microphone signal into one-second windows and averaged all the windows in the time domain to remove unwanted noise.
4. We performed Fast Fourier Transform (FFT) on the averaged temporal impulse response to get the frequency response of the system.

To compensate for the possible unevenness in the frequency responses of the microphone and loudspeaker, we measured the frequency response of the loudspeaker in free air with the same microphone, using the same protocol. Then we divided the frequency responses of the subglottal tracts by the frequency response of the loudspeaker obtained in free air. We used Matlab custom-made scripts to perform all the numerical computations. The resonant subglottal tract was manually set to the previously determined piston positions corresponding to *f*_*R1*_ = 330, 400, 500, 600, 700 and 800 Hz.

### Excised larynx experiments

#### Preparation of the larynges

During the excised larynx experiments we used red deer (*cervus elaphus*) larynges. These larynges were shown to behave similarly as human larynges^54,55^. The larynges were harvested from animals living wildly in forests, which were hunted by the Czech Army Forest Service during a regular hunting season, and they were treated in accordance with the standard ethical requirements of the Palacký University in Olomouc. After being harvested, the larynges were ‘flash-frozen’ using liquid nitrogen and kept in a freezer. Before the experiment, the larynges were put in a water bath heated to 30 °C until the larynx was completely defrosted. We prepared the larynges to expose the vocal folds, by removing the tissues above them: the epiglottis, the ventricular folds and part of the thyroid cartilage^56–58^. We attached the larynges at the open end of the subglottal tract and tightened them to prevent air leaks.

#### Experimental procedure

We used data from two separate experiments with red deer larynges. During these experiments, metal prongs were used to adduct the vocal folds and keep the laryngeal adjustment constant.

- In the first experiment (steady phonations), we adjusted the air flow to approximately 400 mL/s and waited until the subglottal pressure stabilized to a final value. After getting representative data we stopped the flow. We first performed the experiment with the anechoic subglottal tract attached to the system, then with the resonant subglottal tract set to six different values of *f*_*R1*_: 330, 400, 500, 600, 700 and 800 Hz. To rule out the possibility of the long-term laryngeal tissue changes influencing the results, we then repeated the procedure once again with the anechoic subglottal tract and with the resonant subglottal tract set to the same values of *f*_*R1*_, in order to verify the repeatability of the results.
- During the second experiment (flow sweeps), we first used the anechoic subglottal tract, and performed three flow sweeps, each executed in the following manner: the flow was slowly increased from 0 to approximately 550 mL/s, and after about five seconds we slowly decreased the flow back to 0 mL/s, for a total duration of about one minute per sweep. After that, we changed the subglottal tract to the resonant one (set to *f*_*R1*_ = 500 Hz), and repeated the three flow sweeps with the same flow values. Then, we switched back to the anechoic subglottal tract, and repeated the whole experiment once again to verify the repeatability of the results. Four larynges were used during the experiment, but one of them could not vibrate steadily, therefore we decided to discard it.

#### Data processing

The fundamental frequency *f*_*o*_ was estimated with the SWIPE’ algorithm developed by Camacho et al.^59^. The onset and offset threshold pressures were measured by manually finding the oscillation onsets and offsets in the subglottal pressure waveform. We used a custom Matlab script to click on the waveform and get the onset and offset times. Afterwards, we averaged the subglottal pressure over a 50 ms window before the onset and after the offset. The radiated SPL was derived from the calibrated microphone signal at 10 cm from the vocal folds. We used the SPL calibration method 1A (using a calibrator and the microphone) described by Švec & Granqvist^50^ together with the corresponding software package for Matlab^60^. The subglottal SPL was derived from the calibrated subglottal pressure signal, measured by the pressure sensor placed just below the vocal folds. The subglottal pressure was calibrated in cm H_2_O using a U-shaped tube. The pressures were converted to hectopascals by multiplying it by the factor of 0.981. We applied a high-pass filter with a cutoff frequency of 10 Hz to the subglottal pressure and microphone signals to remove any DC offset, and then calculated both SPLs on the filtered signals using ‘fast’ time-weighting and no frequency weighting.

### Inverse filtering

We performed inverse filtering analysis using the Sopran software developed by Svante Granqvist^33^. The signals were downsampled to 6 kHz to make the procedure easier. Through the software one can manually set up inverse poles and zeros to obtain a waveform as close to the theoretical source signal waveform as possible. Sopran also includes an option to select the type of the original signal: ‘Sound pressure (mic)’ or ‘Flow signal (mask)’. When the first option is selected, Sopran adds a numerical integration step and a high-pass filter, for which the cutoff frequency is manually set by the user. As we used the microphone signal registering the radiated sound pressure, we accordingly selected the ‘Sound pressure (mic)’ option and used the cutoff frequency 20 Hz.

### Statistics

Statistical tests were performed to find out significance of the differences between the anechoic and resonant subglottal conditions in the phonation onset and phonation offset pressures. Since we had onset and offset pressure data from two repetitions of three pressure sweeps for three larynges in both anechoic and resonant conditions, we utilized linear regression models with multiple categorical variables to take all these factors into account. We performed the statistical tests using Matlab built-in functions. The details on the statistical treatment of our data are provided in the Supplementary materials S1 to this article.

### Supplementary information

Supplementary Information

## Data Availability

The datasets generated and/or analyzed during the current study are available from the corresponding author on request.

## Appendix

### Theory of the relationship between the anechoic subglottal pressure, the glottal flow modulation and the radiated sound pressure

As mentioned in the methods section, the subglottal tract has the shape of a straight circular waveguide, and in the anechoic conditions it is assimilated to a semi-infinite waveguide. We can therefore assume that plane waves propagate in this waveguide in only one direction along the longitudinal axis of the waveguide. If we now consider the upwards direction (towards the glottis) along this axis to be the positive direction, then the subglottal acoustic waves propagate in the negative direction (away from the glottis). Below the glottis, the subglottal acoustic pressure $p_{s}$ decreases when the glottis opens and increases when it closes, while the subglottal acoustic velocity $u_{s}$ increases when the glottis opens and decreases when the glottis closes. This means $p_{s}$ and $u_{s}$ have an opposite phase. Furthermore, since in the anechoic conditions the waves propagate in only one direction, the amplitudes of $p_{s}$ and $u_{s}$ are proportionally related through the characteristic impedance $Z_{c} = \rho_{0} c$^26^, where $\rho_{0}$ is the mean density of air and $c$ the velocity of sound in air. It is therefore possible to write the relation between both quantities:

1 $$u_{s} = - \frac{{p_{s} }}{{\rho_{0} c}} = - \frac{{p_{s} }}{{Z_{c} }} .$$

For moist air with the temperature of 37 °C, it holds $Z_{c} \approx 400$ Pa∙s∙m^-1^. If we express the subglottal volume flow $U$ as the particle velocity $u_{s}$ multiplied by the cross-sectional area of the anechoic subglottal tract $S$ (in our case $S = 4.5$ mm^2^), the volume flow can be expressed as:

2 $$U = - \frac{S}{{Z_{c} }}p_{s} .$$

Since $S$ and ${\text{Z}}_{{\text{c}}}$ are constants, the equation justifies that the acoustic pressure and the acoustic flow are proportional inside the anechoic subglottal tract and their waveform shapes are identical, but with reversed polarity.

The radiated acoustic pressure can be estimated from the oscillatory glottal volume flow. Because the glottal area is small compared to the wavelengths of the dominant frequencies of the voice spectrum, it is reasonable to approximate the oscillating glottis as a point source, generating the glottal volume flow $Q$. In the frequency domain, the glottal volume flow can be expressed as $Q = Q_{0} \left( \omega \right)e^{j\omega t}$, where $Q_{0} \left( \omega \right)$ is the amplitude, $j^{2} = - 1$, and $\omega$ is the angular frequency. Here $Q$ only corresponds to the acoustic component of the glottal flow, disregarding the steady component which does not contribute to the sound. In this case, the radiated acoustic pressure $p_{r}$, in free air at the distance $r$ from the glottis and at the angular frequency $\omega$, can be estimated from the time derivative of the glottal volume flow by the theoretical relationship^61^:

3 $$p_{r} \left( {r,\omega } \right) = \frac{{\rho_{0} }}{4\pi r}\frac{\partial Q}{{\partial t}} = \frac{{j\omega \rho_{0} Q}}{4\pi r}.$$

The complex notation is only useful here to know the phase difference (here 90 degrees) between the flow and the pressure. It is possible to express the volume flow $U$ and the acoustic pressure $p_{s}$ in the frequency domain through the Fourier series, as a sum of sinusoidal components of different amplitude, phase and frequency. If we assume that for every angular frequency $\omega$, the subglottal volume flow $U\left( \omega \right)$ is equal to the glottal flow $Q\left( \omega \right)$, combination of Eqs. (2) and (3) provides an analytical relation between the anechoic subglottal pressure $p_{s}$ and the radiated sound pressure $p_{r}$:

4 $$p_{r} \left( {r,\omega } \right) = - \frac{{j\omega \rho_{0} S}}{{4\pi rZ_{c} }}p_{s} = - jA\left( {r,\omega } \right)p_{s} \left( \omega \right),$$

where $A\left( {r,\omega } \right) = \frac{{{{\omega \rho }}_{0} {\text{S}}}}{{4{{\pi rZ}}_{{\text{c}}} }}$. To find the sound pressure level (SPL) difference between the radiated and subglottal sound in decibels, we can use the amplitudes of the radiated and subglottal pressure components ($p_{rA} \left( \omega \right)$ and $p_{sA} \left( \omega \right)$, respectively) and express their ratio logarithmically as:

5 $$A_{L} \left( \omega \right) = 20\log \frac{{p_{rA} \left( \omega \right)}}{{p_{sA} \left( \omega \right)}} = 20\log \frac{{\omega \rho_{0} S}}{{4\pi rZ_{c} }},$$

The theoretical dependence of $A_{L}$ on frequency, for the distance $r = 10$ cm, was plotted by solid line in Fig. 8 for frequencies ranging from 50 to 2000 Hz.
