# Supplementary material for: Subglottal pressure oscillations in anechoic and resonant conditions and their influence on excised larynx phonations
Source: Sci Rep. 2021 Jan 8;11:28. doi: 10.1038/s41598-020-79265-3 (PMC7794390; doi:10.1038/s41598-020-79265-3)
Supplement: Supplementary file 1 — Supplementary Information [file 41598_2020_79265_MOESM1_ESM.pdf]

Title: Subglottal pressure oscillations in anechoic and resonant conditions and their influence on excised larynx phonations

Sarah Lehoux[1], Vít Hampala[1], Jan G. Švec[1]

[1]Voice Research Lab, Department of Biophysics, Faculty of Science, Palacký University, Olomouc, Czechia

sarah.h.lehoux@gmail.com, vit.hampala@gmail.com, jan.svec@upol.cz

Corresponding author:

Jan G. Švec (jan.svec@upol.cz), Department of Biophysics, Faculty of Science, Palacký University, 17. Listopadu 12, 771 46 Olomouc, Czechia

Supplementary materials

Statistical analysis of the phonation onset and offset pressures

Phonation onset and phonation offset pressures were measured for three larynges in anechoic and resonant conditions using pressure sweeps. For each larynx, we always performed, at first, 3 pressure sweeps in anechoic conditions, then 3 sweeps in resonant conditions, then we repeated the 3 sweeps in anechoic conditions and, at last, we repeated the 3 sweeps in resonant conditions. For each individual sweep, we measured the onset and offset phonation threshold pressures. The resulting measured values are listed in Table S1.

Larynx number	Onset pressure (hPa)		Offset pressure (hPa)		Flow sweep number
	Anechoic	Resonant	Anechoic	Resonant	
Larynx #1	6.55	5.91	4.74	4.55	1a
	5.91	6.29	5.09	4.58	1b
	5.58	6.61	5.01	4.53	1c
	6.03	6.14	5.16	4.50	2a
	6.21	6.51	5.09	4.70	2b
	6.27	6.17	5.19	4.59	2c
Larynx #2	7.16	7.03	4.93	5.19	1a
	5.65	5.97	5.94	5.23	1b
	5.41	5.89	6.61	4.71	1c
	7.09	6.14	7.21	5.55	2a
	5.91	6.21	6.85	5.68	2b
	6.12	5.76	5.52	5.27	2c
Larynx #3	5.58	5.84	3.22	2.24	1a
	4.42	3.93	3.18	2.51	1b
	4.35	3.67	3.03	2.58	1c
	5.51	5.92	2.91	2.82	2a
	3.88	4.07	2.69	2.99	2b
	3.99	3.78	2.66	2.98	2c

Supplementary Table S1. Measured values of the onset and offset pressures, for the anechoic and resonant conditions. The ‘Flow sweep number’ column gives the chronological order in which each measure was done: 1x (where x = a, b or c) correspond to the three flow sweeps done in the first series of measurements and 2x to the three flow sweeps done in the repeated series of measurements, for each larynx.

Our main goal was to find out whether the resonance conditions influence the phonation threshold pressures. For statistical testing, we separated the onset and offset pressures and treated them independently. Besides the resonance conditions (anechoic versus resonant), we needed to consider two more factors that could have influenced the results – the larynx (no. 1, 2, 3) and the repetition

(series 1 and series 2). To investigate the influence of all these factors, including their interactions, we chose to use a linear regression model with categorical variables. This model tests the dependency of the threshold pressures p_{th} on all the factors in the basic form:

$$p_{th} \approx 1 + Condition + Larynx + Series + Condition * Series + Larynx * Series + Condition * Larynx \quad \text{(S1)}$$

Results for the offset thresholds:

At first, we applied the model on the offset pressures. The offset pressures for the different larynges, conditions and series are summarized in Figure S1. The model analysis yielded the three interaction terms from eq. S1 to be insignificant (Condition*Series: p = 0.55, Larynx*Series: p = 0.22, Condition*Larynx: p = 0.14), allowing them to be removed from the model.

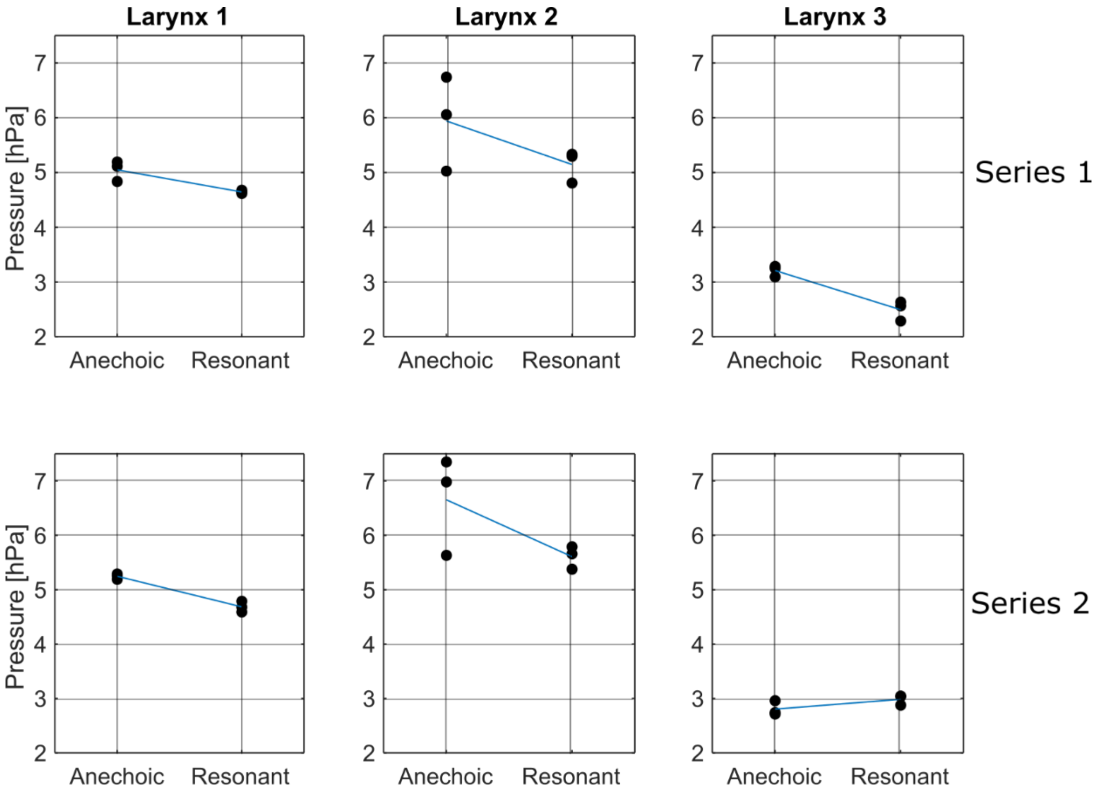

Supplementary Figure S1. Phonation offset pressure values (black dots) for each larynx and the two repeated series of measurements. The blue lines indicate linear regressions.

Applying the regression model without the interaction terms:

$$p_{th} \approx 1 + Condition + Larynx + Series \tag{S2}$$

yielded the Series factor to be insignificant (p = 0.08). This indicated that the differences between the results from the two repeated series of measurements could be neglected. Based on this finding, we pooled the values from the two series together.

The pooled results are shown in Figure S2. Comparison of the medians indicate that, in all the three larynges, the pressures are larger in anechoic than in resonant conditions. Nevertheless, the results differ among the three larynges. There is a trend of larger differences for the larynges with larger values of the pressures. This is indicated by the red lines in Figure S2. Notice, that the Larynx 2, which vibrated at the largest pressures, shows also the largest differences between the anechoic and resonant conditions, whereas the smallest differences between anechoic and resonant conditions are found for the Larynx 3, which vibrated at the lowest pressures. This suggests that, instead of the pressure differences, it is more appropriate to study the pressure ratios between the anechoic and resonant conditions. Table S2 shows that these ratios are much more similar across the three larynges (0.85-0.91) than the differences (0.27-1.02). To study the ratios, we apply the linear regression model on the logarithm of the measured pressures (hence, we assume that the pressures follow the log-normal distribution).

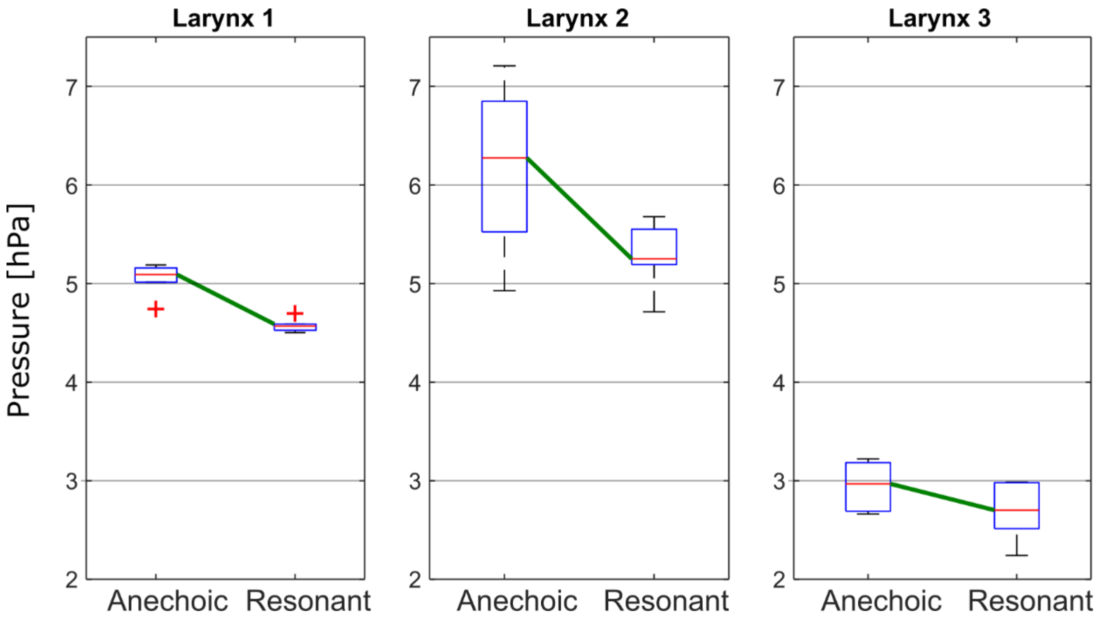

Supplementary Figure S2. Boxplots of the offset pressure values for the three larynges and the two resonance conditions. The green lines connect the medians (red horizontal lines) of the measured values and indicate the average difference between the anechoic and resonant conditions in each larynx. The blue boxes extend from the 25th to the 75th percentiles, the whiskers extend to the most extreme data points (not included outliers), and the red crosses indicate the outliers.

	Larynx 1	Larynx 2	Larynx 2
Anechoic	5.09	6.27	2.97
Resonant	4.57	5.25	2.70
Difference	0.42	1.02	0.27
Ratio	0.91	0.85	0.91

Supplementary Table S2. Median values of the offset pressures [hPa] for each larynx, together with their differences and their ratios.

Since we converted the input data to the logarithms, we apply the linear regression model to the logarithmic data again in the full form:

$$log\ (p_{th}) \approx 1 + Condition + Larynx + Series + Condition * Series + Larynx * Series + Condition * Larynx \quad \text{(S3)}$$

Here again, the three interaction terms as well as the Series term show up to be insignificant (Condition*Series: p = 0.13, Larynx*Series: p = 0.43, Condition*Larynx: p = 0.65, Series: p = 0.08), similarly as in the non-logarithmic version of the model. Therefore, we utilize the final reduced model:

$$log\ (p_{th}) \approx 1 + Condition + Larynx \quad \text{(S4)}$$

This model yields the estimated coefficients and p-values as listed in the Table S3.

	Estimate	Standard error	t-statistic	p-value
Intercept	0.7150	0.0124	57.8	<0.0001
Condition_Resonant	-0.0498	0.0124	-4.0	0.0003
Larynx_2	0.0725	0.0152	4.8	<0.0001
Larynx_3	-0.2338	0.0152	-15.4	<0.0001

Supplementary Table S3. Results obtained for the offset threshold pressures using the linear regression model (S4)

These results reveal that there are highly significant differences in the offset pressures between the anechoic (taken as reference) and resonant conditions (p = 0.0003), between Larynx 1 and Larynx 2 (p < 0.0001), and between Larynx 1 and Larynx 3 (p < 0.0001). The coefficient for the resonant versus anechoic condition is estimated by the model at the value of C_{log} = -0.050 ± 0.012. Doubling the standard error yields the 95% confidence interval for this coefficient C_{log_95CI} = (-0.075, -0.025).

Finally, let us transform the logarithmic coefficient C_{log} to the ratio C to find out how much the resonance conditions influence the resulting offset pressures. It holds

$$log(p_{resonant}) - log(p_{anechoic}) = C_{log} = log(C)$$
$$C = \frac{p_{resonant}}{p_{anechoic}} = 10^{C_{log}} \quad \text{(S5)}$$

From there, we obtain the (median) value of the coefficient *C* = 0.89 and its 95% confidence interval (0.84, 0.94). These results reveal that the resonant subglottal tract lowers the offset threshold pressures on average by 11%, with the 95% confidence interval being between 6% and 16%.

Results for the onset thresholds:

The onset threshold pressure values for the three larynges, the two resonance conditions and the two series (repetitions) are summarized in Figure S3. For the statistical testing, we applied the same procedures as for the offset data. Again, we assumed the log-normal distribution of the pressures and therefore the linear regression models were applied to the logarithms of the pressures.

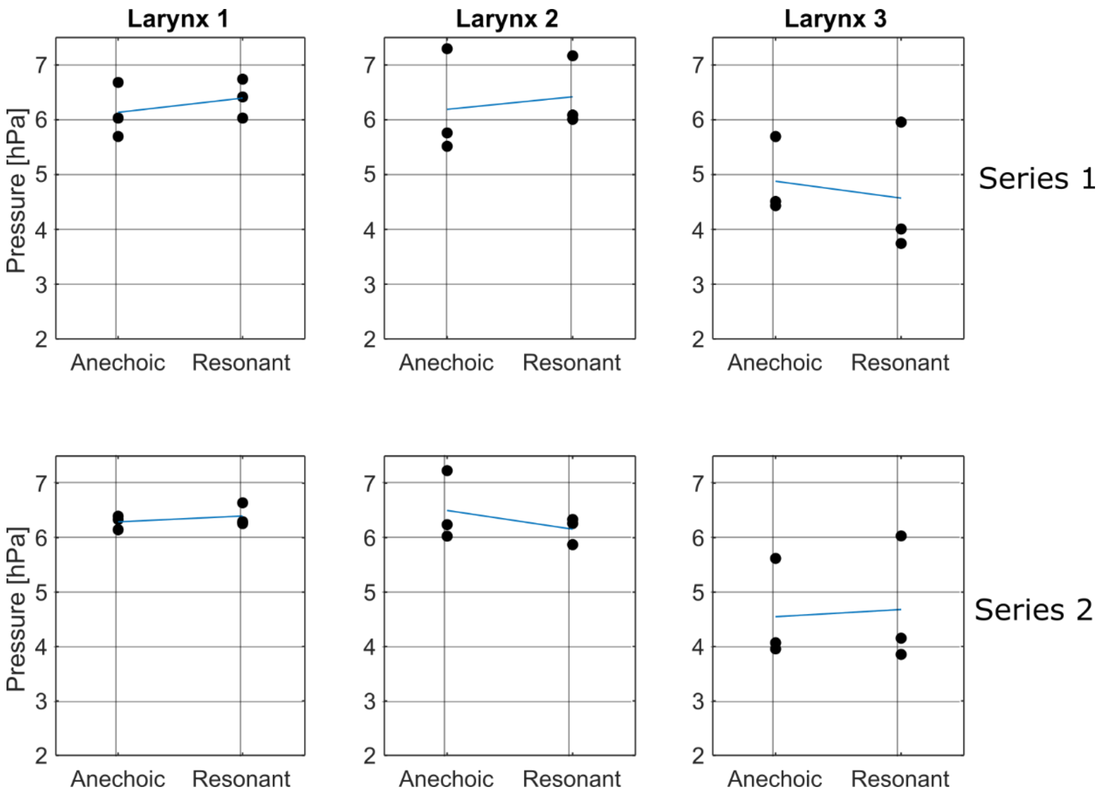

Supplementary Figure S3. Phonation onset pressure values (black dots) for each larynx and the two repeated series of measurements. The blue lines indicate linear regressions.

Similarly to the offset case, all the interaction terms as well as the Series factor were found insignificant ($p = 0.87$ for the Larynx*Conditions interaction; $p = 0.95$ for the Series*Conditions interaction; $p = 0.93$ for the Larynx*Series interaction; $p = 0.98$ for the Series factor) and could be removed. The reduced model

$$log\ (p_{th}) \approx 1 + Condition + Larynx \quad \text{(S6)}$$

yielded the results as listed in Table S4, revealing the significant difference being only between the larynx 1 (taken as the reference) and larynx 3 ($p < 0.0001$). Importantly, these results reveal that the resonant subglottal tract did not influence the phonation onset pressures significantly ($p = 0.97$) in our experiment when compared to the anechoic conditions. This contrasts with the significant effect observed on the offset pressures. We can therefore conclude that the resonant subglottal tract influenced the onset and offset pressures differently.

	Estimate	Standard error	t-statistic	p-value
Intercept	0.7997	0.0179	44.707	< 0.0001
Condition_Resonant	-0.0008	0.0179	-0.042	0.9669
Larynx_2	-0.0005	0.0219	-0.021	0.9837
Larynx_3	-0.1370	0.0219	-6.249	< 0.0001

Supplementary Table S4. Results obtained for the onset threshold pressures using the linear regression model (S6).
